# Prediction of Sepsis in the Intensive Care Unit With Minimal Electronic Health Record Data: A Machine Learning Approach

**DOI:** 10.2196/medinform.5909

**Published:** 2016-09-30

**Authors:** Thomas Desautels, Jacob Calvert, Jana Hoffman, Melissa Jay, Yaniv Kerem, Lisa Shieh, David Shimabukuro, Uli Chettipally, Mitchell D Feldman, Chris Barton, David J Wales, Ritankar Das

**Affiliations:** ^1^ Dascena, Inc Hayward, CA United States; ^2^ Department of Clinical Informatics Stanford University School of Medicine Stanford, CA United States; ^3^ Department of Emergency Medicine Kaiser Permanente Redwood City Medical Center Redwood City, CA United States; ^4^ Department of Medicine Stanford University School of Medicine Stanford, CA United States; ^5^ Division of Critical Care Medicine Department of Anesthesia and Perioperative Care University of California San Francisco San Francisco, CA United States; ^6^ Department of Emergency Medicine Kaiser Permanente South San Francisco Medical Center South San Francisco, CA United States; ^7^ Department of Emergency Medicine University of California San Francisco San Francisco, CA United States; ^8^ Division of General Internal Medicine Department of Medicine University of California San Francisco San Francisco, CA United States; ^9^ Department of Chemistry University of Cambridge Cambridge United Kingdom

**Keywords:** sepsis, machine learning, clinical decision support systems, electronic health records, medical informatics

## Abstract

**Background:**

Sepsis is one of the leading causes of mortality in hospitalized patients. Despite this fact, a reliable means of predicting sepsis onset remains elusive. Early and accurate sepsis onset predictions could allow more aggressive and targeted therapy while maintaining antimicrobial stewardship. Existing detection methods suffer from low performance and often require time-consuming laboratory test results.

**Objective:**

To study and validate a sepsis prediction method, *InSight*, for the new Sepsis-3 definitions in retrospective data, make predictions using a minimal set of variables from within the electronic health record data, compare the performance of this approach with existing scoring systems, and investigate the effects of data sparsity on *InSight* performance.

**Methods:**

We apply *InSight*, a machine learning classification system that uses multivariable combinations of easily obtained patient data (vitals, peripheral capillary oxygen saturation, Glasgow Coma Score, and age), to predict sepsis using the retrospective Multiparameter Intelligent Monitoring in Intensive Care (MIMIC)-III dataset, restricted to intensive care unit (ICU) patients aged 15 years or more. Following the Sepsis-3 definitions of the sepsis syndrome, we compare the classification performance of *InSight* versus quick sequential organ failure assessment (qSOFA), modified early warning score (MEWS), systemic inflammatory response syndrome (SIRS), simplified acute physiology score (SAPS) II, and sequential organ failure assessment (SOFA) to determine whether or not patients will become septic at a fixed period of time before onset. We also test the robustness of the *InSight* system to random deletion of individual input observations.

**Results:**

In a test dataset with 11.3% sepsis prevalence, *InSight* produced superior classification performance compared with the alternative scores as measured by area under the receiver operating characteristic curves (AUROC) and area under precision-recall curves (APR). In detection of sepsis onset, *InSight* attains AUROC = 0.880 (SD 0.006) at onset time and APR = 0.595 (SD 0.016), both of which are superior to the performance attained by SIRS (AUROC: 0.609; APR: 0.160), qSOFA (AUROC: 0.772; APR: 0.277), and MEWS (AUROC: 0.803; APR: 0.327) computed concurrently, as well as SAPS II (AUROC: 0.700; APR: 0.225) and SOFA (AUROC: 0.725; APR: 0.284) computed at admission (*P*<.001 for all comparisons). Similar results are observed for 1-4 hours preceding sepsis onset. In experiments where approximately 60% of input data are deleted at random, *InSight* attains an AUROC of 0.781 (SD 0.013) and APR of 0.401 (SD 0.015) at sepsis onset time. Even with 60% of data missing, *InSight* remains superior to the corresponding SIRS scores (AUROC and APR, *P*<.001), qSOFA scores (*P*=.0095; *P*<.001) and superior to SOFA and SAPS II computed at admission (AUROC and APR, *P*<.001), where all of these comparison scores (except *InSight*) are computed without data deletion.

**Conclusions:**

Despite using little more than vitals, *InSight* is an effective tool for predicting sepsis onset and performs well even with randomly missing data.

## Introduction

Sepsis and its associated syndromes are among the leading causes of worldwide morbidity and mortality [1] and are responsible for placing an enormous cost burden on the health care system [2]. Sepsis, severe sepsis, and septic shock are umbrella terms for a broad and complex variety of disorders characterized by a dysregulated host response to infectious insult. Because of the heterogeneous nature of possible infectious insults and the diversity of host response, these disorders have long been difficult for physicians to recognize and diagnose. A redefinition of sepsis has been recently introduced with the goal of increasing the accurate identification of septic patients in clinical and preclinical settings. This new definition, Sepsis-3 [3], eliminates the traditional ternary classification of sepsis progression from sepsis, through severe sepsis, to septic shock and instead utilizes a two-tier identification system tied to increases in mortality probability. Under the new definition, the term “sepsis” is defined as a “life-threatening organ dysfunction caused by a dysregulated host response to infection [3],” which corresponds most closely with the previously established definition of severe sepsis. Organ dysfunction is defined in practice as an increase in the Sequential Organ Failure Assessment (SOFA) [4] score of at least 2 points. These parameters are associated with in-hospital mortality above 10%. Singer et al [3] define “septic shock” as a classification of sepsis “in which underlying circulatory and cellular metabolism abnormalities are profound enough to substantially increase mortality,” and suggest identifying such patients by a serum lactate measurement above 2 mmol/L and hypotension requiring administration of vasopressors to maintain a mean arterial pressure above 65 mm Hg. Septic shock conditions are associated with in-hospital mortality over 40%. We use this newly proposed definition for sepsis as a gold standard for the implementation of our predictive algorithm, *InSight* [5,6]. *InSight* uses only 8 common measurements (vital signs and other easily assessed bedside measurements, plus age) obtained from electronic health records (EHRs) for the prediction and detection of sepsis in the intensive care unit (ICU) population.

A new bedside scoring system to be used outside the ICU, “qSOFA” (for “quick SOFA”), has been proposed as a screening mechanism to prompt the clinician to further investigate for sepsis or to transfer to a higher level of care [3]. The criteria for qSOFA are at least 2 of the following: respiration above 22/min, altered mentation, or systolic blood pressure below 100 mm Hg. Other scoring systems in current use for the determination or prediction of sepsis include the SOFA score [4], the Modified Early Warning Score (MEWS) [7], the Simplified Acute Physiology Score (SAPS II) [8], and Systemic Inflammatory Response Syndrome (SIRS) criteria [9]. These methods utilize tabulation of various patient vital signs and laboratory results to generate risk scores; however, they do not analyze trends in patient data or correlations between measurements.

The purpose of this study is to validate the *InSight* sepsis prediction method for the new Sepsis-3 definitions using retrospective data consisting of minimal, commonly available EHR variables, and to investigate the effects of data sparsity on its performance. In addition, *InSight* predictive performance will be compared with other existing scores and systems.

## Methods

### Dataset

This work uses the Multiparameter Intelligent Monitoring in Intensive Care (MIMIC)-III version 1.3 dataset [10], compiled from the Beth Israel Deaconess Medical Center (BIDMC) in Boston, MA between 2001 and 2012. The MIMIC-III set includes anonymized data from over 52,000 ICU stays and more than 40,000 patients. The *InSight* algorithm uses only the EHR-entered components of the MIMIC-III set, and does not require real-time waveform data or the interpretation of free text notes. The MIMIC-III set includes data logged using the CareVue (Philips) and Metavision (iMDSoft) EHR systems, which handle and store some pieces of information differently. These systems were used at BIDMC from 2001 to 2008 and 2008 to 2012, respectively. Since the original MIMIC-III data collection did not impact patient safety and all data were deidentified in accordance with the Health Insurance Portability and Accountability Act (HIPAA) Privacy Rule, the requirement for patient consent was waived by the Institutional Review Boards of BIDMC and the Massachusetts Institute of Technology.

### Data Extraction and Imputation

We collect a variety of data from the MIMIC-III dataset to define sepsis onset and calculate the *InSight* score, as well as other scores such as MEWS and SOFA for comparison. All data are extracted from the MIMIC-III set using custom PostgreSQL (PostgreSQL Global Development Group) queries. These measurements are temporally binned using a bin width of one hour; the measurement values are then averaged within a bin. This process and all subsequent calculations are carried out in MATLAB (The Mathworks, Natick, MA). Missing data are imputed using a “carry-forward” system, where the most recent bin value is carried forward to fill subsequent empty bins. In order to provide a comparison not confounded by different data availability at different times preonset, bins that precede the collection of any measurements of the corresponding type are back-filled with the value of the first subsequent bin with measurements. These processed data are then used in downstream calculations.

### Gold Standard

We follow the sepsis definition promulgated by Singer et al [3]. Specifically, Singer et al define sepsis as “life-threatening organ dysfunction caused by a dysregulated host response to infection ... [signified by] an acute change in total SOFA score ≥2 points consequent to the infection.” Following the retrospective validation study of Seymour et al [11], we retrospectively equate suspicion of infection with an order for a culture lab draw, together with a dose of antibiotics, within a specified window (see Table 1). Due to limitations of the latest release of MIMIC-III (v1.3), negative cultures (blood and other types) are underreported in the database.

To identify an acute change in SOFA score, we adhere to the definition proposed by Seymour et al. Taking the initial time of the earliest culture draw or antibiotic administration as the time of suspicion of infection, we define a window of up to 48 hours before this time (limited by time of data availability) and 24 hours after this time (limited by time of departure from the ICU). The SOFA score at the beginning of this window is compared with its hourly value throughout this window; if this hourly value is ≥ 2 points higher than the value at the start of the window, we define the first such hour as the onset of sepsis and designate the patient as septic (class 1). If a patient fails to have such an event, we classify them as nonseptic (class 0). If the data required to calculate one of the SOFA subscores is not present in the imputed data, that subscore is given the value 0 (ie, “normal”). We also use a modified version of the SOFA respiration score [12], which avoids requiring information regarding patient mechanical ventilation. Seymour et al were primarily concerned with large-scale identification of septic patients, rather than specifically pinpointing *when* these patients became septic. In contrast, we require this temporal information because we are studying a system that *anticipates* the onset of sepsis.

**Table 1:** Windows of suspected infection, as defined by the presence of a culture and antibiotic administration, following Seymour et al [11].

First event	Window in which second event must occur
Antibiotics administered	Culture taken in the following 72 hours
Culture taken	Antibiotics administered in the following 24 hours

### Selected Clinical Measurements and Patient Inclusion

**Figure 1:**
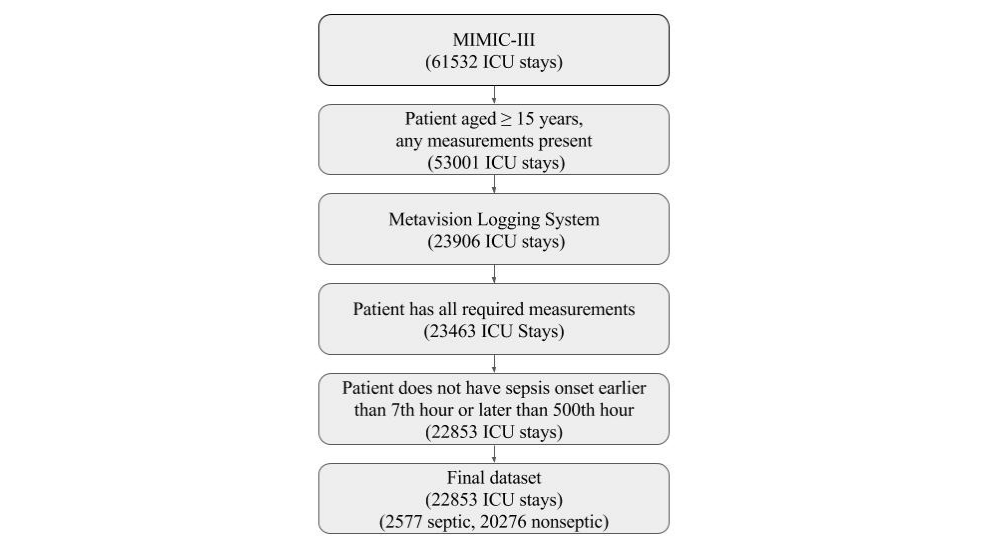
Inclusion diagram. All intensive care unit (ICU) stays meeting the sequential inclusion criteria outlined above are included in the training and testing sets. The final dataset has a sepsis prevalence of 11.3%. MIMIC-III: Multiparameter Intelligent Monitoring in Intensive Care version III.

The learning method employed by *InSight* is flexible with regard to the patient data it uses. For the present work, we have selected systolic blood pressure, pulse pressure, heart rate, respiration rate, temperature, peripheral capillary oxygen saturation (SpO_2_), age, and Glasgow Coma Score (GCS). All of these features are nearly universally available at the bedside and do not rely on laboratory tests. There is disagreement about which patient measurements constitute vital signs with the most restrictive definitions only including temperature, heart rate, blood pressure, and respiratory rate, and the most inclusive ones including all of the patient data used in this study with the exception of age [13,14]. Thus, we have collectively labeled the set of measurements used in this study as “extended vitals.” Although we train and test our method in the ICU, we note that these or similar features should also be available in other settings. Successful prediction from a minimal set of extended vital signs allows for general application of our approach. This feature is particularly useful for patients that cannot be assessed using other scoring systems (eg, SOFA). We exclude all ICU stays from consideration if any of the following are true: the patient was not at least 15 years old (to eliminate pediatric patients); no measurements were recorded in the ICU; the ICU data was logged using CareVue, rather than Metavision; one or more of the measurements required for our predictor were not recorded at any time during the ICU stay; sepsis onset as defined above occurs, but is more than 500 or less than 7 hours into the ICU stay. The inclusion diagram is presented as Figure 1 and the demographic distribution of patients aged 15 years or more is presented as Table 2. It is important to note that the overall hospital mortality rate of 6.9% for all patients meeting inclusion criteria is significantly lower than the mortality rate for sepsis patients only. This is because the overall study population, as detailed in Table 2, includes patients in all ICU units including low mortality settings like the CSRU. In contrast, the vast majority (over 75%) of infectious disease patients in MIMIC III are in the MICU, which has a median hospital length of stay of 6.4 days and a hospital mortality rate of 14.5% [10].

The requirement that sepsis onset in an included patient occurs be at least 7 hours into their ICU stay is for clarity of presentation. In operation, *InSight* only requires data from the 2 hours preceding prediction time. Given that most patients will have EHR data from a hospital unit that preceded the ICU admission (eg, emergency department, inpatient floor), the predictor will become active at time of admission to the ICU. Notably, the predictor can become active 2 hours after ICU admission at the latest. However, we demonstrate the predictive performance of our approach for various prediction horizons, ie, lengths of time prior to the sepsis onset event. In order for this comparison to not be confounded by differing patient inclusion (varying size and composition) at different horizons, we apply a single, consistent, and conservative inclusion criterion of sepsis onset at least 7 hours into the ICU stay. The requirement that sepsis onset occur within 500 hours (over 20 days) is for convenience of analysis and is minimally restrictive; as shown in Table 2, only 5.1% of patients (1149 patients) have ICU stays of 12 or more days. Similarly, the requirement that all of the chosen measurements are present during the ICU stay is also for analytical convenience, eliminating less than 500 patients, and need not be strictly applied in practice. We plan to loosen this constraint in future work.

The use of only Metavision patients deserves special discussion. For ICU stays logged using the CareVue system, data about procedures performed (ie, cultures being taken) does not appear in the MIMIC-III database in as detailed and comprehensive a fashion as for ICU stays logged using Metavision. Further, while the MIMIC-III version 1.3 dataset includes information from the BIDMC microbiology lab, reporting positive cultures and the results thereof for all patients, negative cultures are not reported consistently. The combination of these facts means that negative cultures are underreported for CareVue patients. This in turn implies that suspicion of infection, as defined by the cooccurrence of culture and antibiotics, is systematically underrepresented in these ICU stays, resulting in a sepsis prevalence of 3.5% for CareVue patients versus 11.3% for Metavision. In light of this disparity, we chose to exclude CareVue patients from our analyses.

We performed an auxiliary analysis to eliminate patients who received antibiotics prior to the start of their ICU stay (4078 of the 23,906 Metavision ICU stays). This was intended to be a highly sensitive, albeit nonspecific way of removing pre-ICU sepsis cases. Since the exact time-stamp of the start of an ICU stay was not available, we approximated it as 60 minutes prior to initial measurement of any of the extended vital signs from the list in the Clinical Measurements section. Although the 60-minute approximation is discussed here, we also examined various other time windows, and the set of excluded patients was not strongly sensitive to the cutoff time used. With the pre-ICU antibiotic removal, the remaining 19,828 ICU stays were screened identically as previously described, leaving a set of 1840 septic ICU stays and 17,214 nonseptic ICU stays (9.66% sepsis prevalence).

**Table 2:** Demographics of the included Multiparameter Intelligent Monitoring in Intensive Care version III (MIMIC-III) intensive care unit stays. All stays correspond to patients aged 15 years or more (21,173 hospital admissions).

Demographic characteristic		Number of ICU Stays n (%)
**ICU type**	medical intensive care unit	9460 (41.89)
	cardiac surgery recovery unit	3345 (14.81)
	surgical intensive care unit	4293 (19.01)
	coronary care unit	2726 (12.07)
	trauma-surgical intensive care unit	2759 (12.22)
**Gender**	Female	9902 (43.85)
	Male	12,681 (56.15)
**Age (years)** Median 65 IQR (53-77)	15-17	25 (0.1)
	18-29	982 (4.3)
	30-39	1132 (5.01)
	40-49	2176 (9.64)
	50-59	4038 (17.88)
	60-69	5159 (22.84)
	70+	9071 (40.17)
**Length of stay (days)** Median 2.0 IQR^a^ (1.2-3.8)	0-2	15,178 (67.21)
	3-5	4267 (18.89)
	6-8	1340 (5.93)
	9-11	649 (2.9)
	12+	1149 (5.09)
**Death during hospital stay**	Yes	1569 (6.95)
	No	21,014 (93.05)

^a^IQR: interquartile range.

### Machine Learning Methods

**Figure 2:**
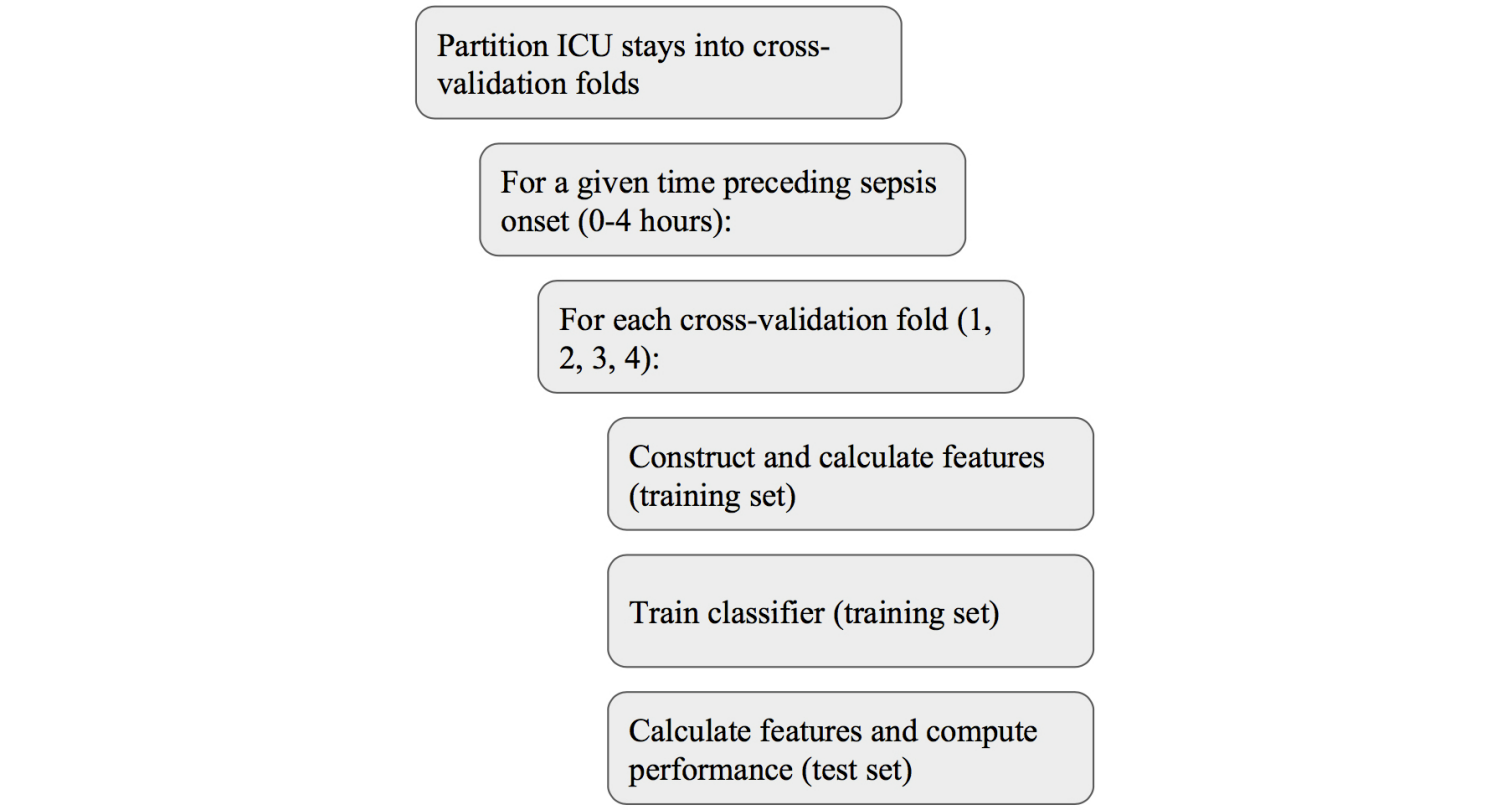
Training and testing procedure. The innermost steps in the process (rightmost) are repeated for each partitioning of the data into cross-validation folds (4 partitionings), for each test cross-validation fold in each partition (4 folds), and each time horizon (5 time horizons). ICU: intensive care unit.

The training and testing process for the *InSight* prediction system consists of 4 stages: data partitioning, feature construction, classifier training, and classifier testing. The entire training and testing procedure is shown diagrammatically in Figure 2. In the first stage, data are partitioned into 4 folds for cross-validation. Each fold is individually used for testing, while the other 3 folds are concatenated to make the corresponding training set. For each cross-validation fold, feature construction is conducted using the training set. Features include the values of the clinical (vital sign) variables chosen for each of the last 2 hours, denoted x_1_ and x_2_; continuous, nonlinear function approximations for each posterior probability of sepsis (s=1) given a smoothed estimate of a single clinical variable x_1_^i^, that is, P(s=1 | x_1_^i^); analogous continuous approximations where Δx^i^ = (x_1_ - x_2_)^i^ is the input, P(s=1 | Δx^i^); and tabular approximations to the posterior probability of sepsis, given combinations of discretized versions of 2 or 3 of the Δx^i^, that is, P(s=1 | Δx^i^, Δx^j^) or P(s=1 | Δx^i^, Δx^j^, Δx^k^). All of these approximations to posterior probabilities of sepsis are calculated exclusively using the training set. The final feature set is:

ξ = [x_1,_ x_2,_ ... P(s=1 | x_1_^i^) ..., ... P(s=1 | Δx^i^) ..., ... P(s=1 | Δx^i^, Δx^j^) ..., ... P(s=1 | Δx^i^, Δx^j^, Δx^k^)... ]

In our first experiment, we assess how performance changes as we use *InSight* to predict whether the patient will become septic at increasingly long times into the future. The *InSight* classifier is given the constructed features and trained to predict whether the patient will be septic (class 1) or not (class 0). This training uses elastic net regularization, which induces a degree of sparsity among the feature weights [15,16]. Finally, the trained classifier is assessed on the disjoint test set; all performance measures presented in this paper are computed on test sets. The entire procedure (fold selection, feature construction, classifier training, and classifier testing) is repeated with independent random partitioning of the data into folds 4 times (ie, 4-fold cross-validation), and for each partitioning, 5 prediction horizons are tested. For each of 0, 1, 2, 3, and 4 hours preceding the time of sepsis onset, we compared *InSight* with qSOFA, MEWS, and SIRS calculated at that time, as well as the SOFA and SAPS II scores computed at ICU admission. While these risk scores are not all sepsis-related, they capture illness severity and represent important benchmarks for performance.

In our second experiment, we test the performance of the *InSight* system in the presence of data sparsity. This situation is simulated by deleting individual EHR-recorded observations according to a random selection procedure. We delete individual observations of the measurements used by our predictor: invasive and noninvasive blood pressure, heart rate, respiration rate, temperature, SpO_2_, and GCS. The frequencies with which these values are recorded in the MIMIC-III database are presented in Table 3. These frequencies are on the order of one measurement per hour, close to our temporal discretization frequency. In our experiments, we require that the first measurement of each type for every ICU stay is retained, but all subsequent measurements for every ICU stay may be deleted uniformly at random with a specified probability of deletion, *P*. We set *P* = {0, .1, .2, .4, and .6} in our experiments. After this random data deletion procedure, we reprocess and impute the data. Note that the gold standard (presence of sepsis and onset time) is determined using the full dataset, and thus is consistent for each ICU stay across all experiments presented here. All subsequent training and testing procedures are similar to the previous experiment.

**Table 3:** Per-hour observation frequencies among included ICU stays (n=22,853). Three ICU stays were of less than 60 minutes and were discarded from these calculations.

Measurement	Mean (SD) (h^-1^)	Median (IQR^a^) (h^-1^)	Fraction of ICU stays (F^b^)
GCS^c^	0.29 (0.16)	0.25 (0.21-0.29)	1
Heart rate	1.31 (3.32)	1.07 (1.01-1.16)	1
Respiration rate	1.30 (3.26)	1.06 (1.00-1.16)	1
SpO_2_^d^	1.27 (3.01)	1.06 (0.99-1.17)	1
Temperature	0.31 (0.21)	0.27 (0.23-0.314)	1
NIDiasABP^e^	0.76 (0.39)	0.88 (0.46-1.02)	0.99
NISysABP^f^	0.76 (0.39)	0.88 (0.46-1.02)	0.99
SysABP^g^	0.41 (1.55)	0 (0-0.76)	0.43
DiasABP^h^	0.41 (1.55)	0 (0-0.76)	0.43

^a^IQR: interquartile range.

^b^F: the fraction of these ICU stays with at least one measurement of the given type.

^c^GCS: Glasgow Coma Score.

^d^SpO_2_: peripheral capillary oxygen saturation.

^e^NIDiasABP: noninvasive diastolic arterial blood pressure.

^f^NISysABP: noninvasive systolic arterial blood pressure.

^g^SysABP: invasive systolic arterial blood pressure.

^h^DiasABP: invasive diastolic arterial blood pressure.

## Results

**Figure 3:**
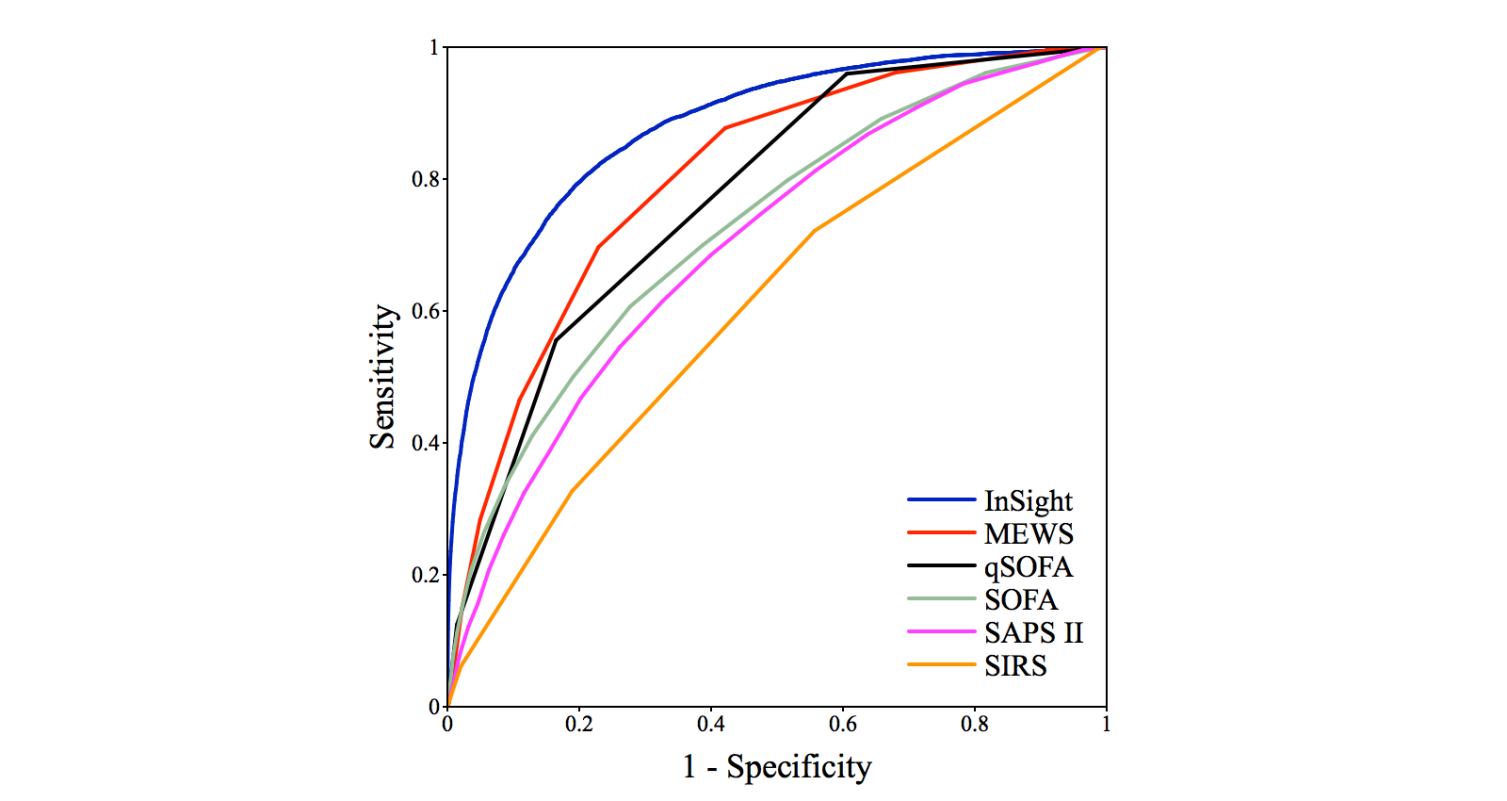
Receiver operating characteristic curves for *InSight* versus competing methods at time of onset. MEWS: Modified Early Warning Score; SOFA: Sequential (Sepsis-Related) Organ Failure Assessment; qSOFA: quick SOFA; SAPS II: Simplified Acute Physiology Score II; SIRS: systemic inflammatory response syndrome.

**Figure 4:**
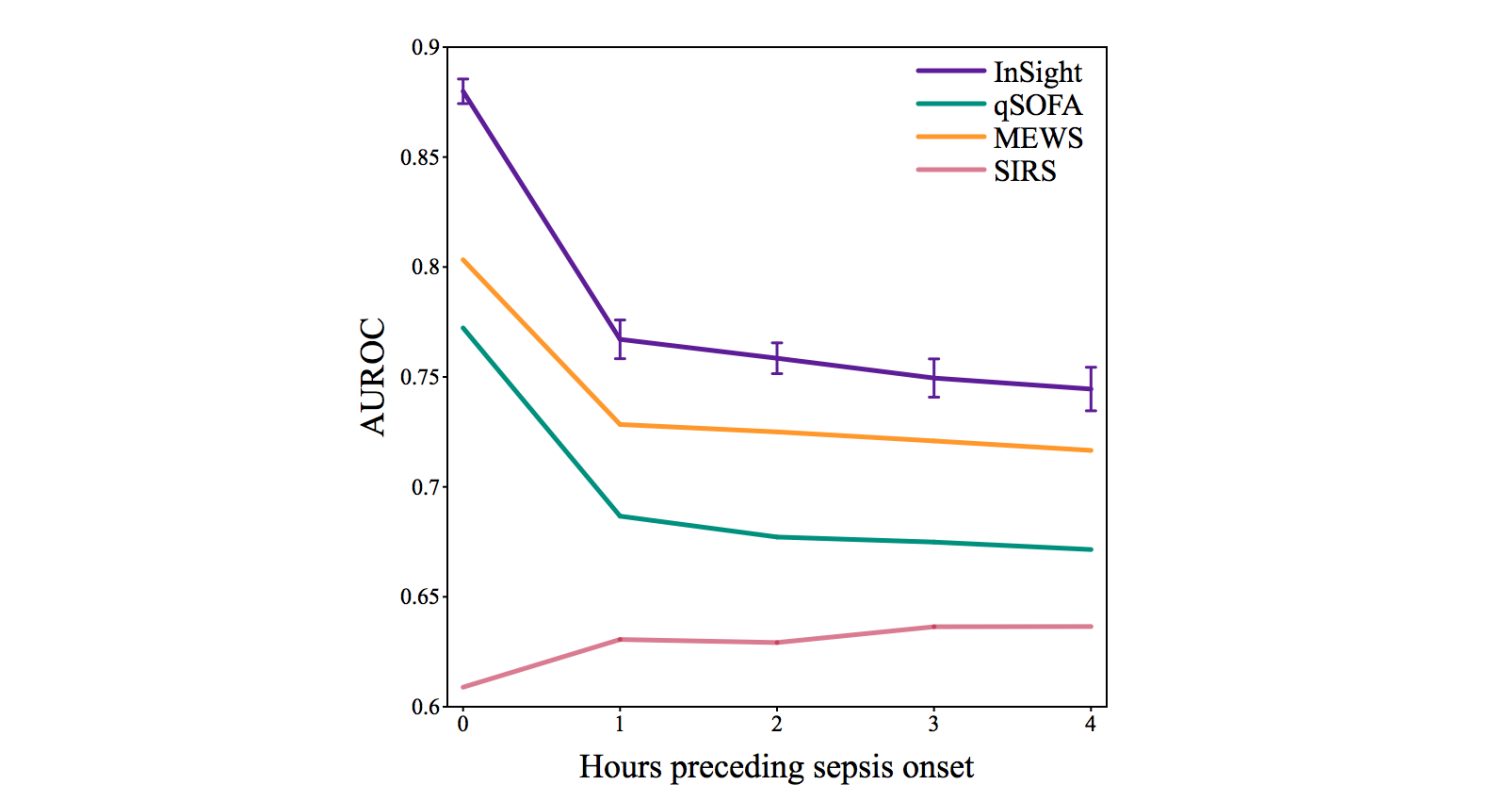
Test set area under receiver operating characteristic curves for *InSight* and competing methods as a function of the amount of time by which prediction precedes potential sepsis onset. Error bars of 1 standard deviation are shown for *InSight*, where the standard deviation is calculated using performance on the cross-validation folds. AUROC: area under the receiver operating characteristic curve; MEWS: Modified Early Warning Score; qSOFA: quick SOFA; SIRS: systemic inflammatory response syndrome.

**Figure 5:**
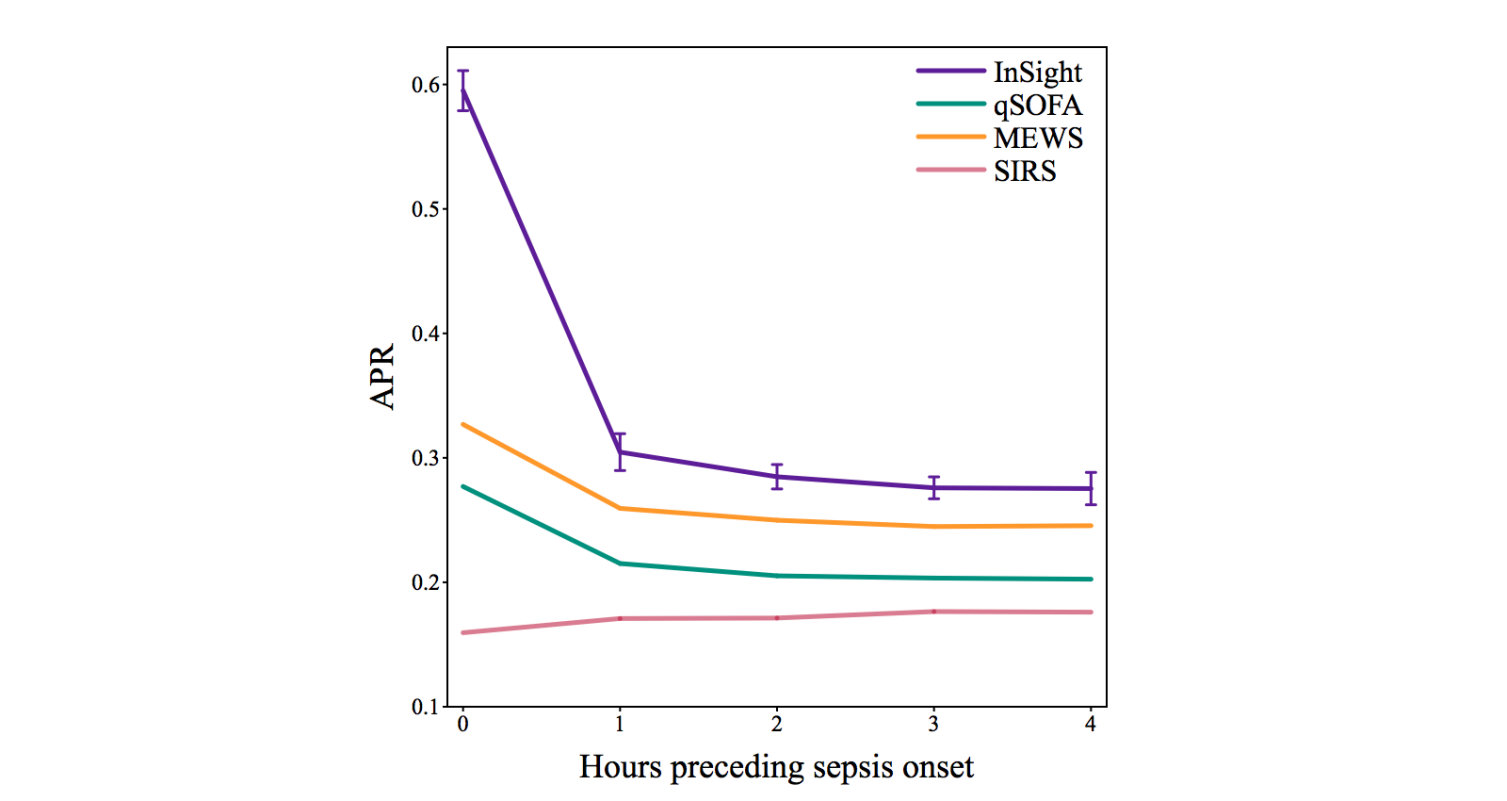
Test set area under precision-recall curves for *InSight* and competing methods as a function of the amount of time by which prediction precedes potential sepsis onset. Error bars of ± 1 standard deviation are shown for *InSight*, where the standard deviation is calculated using performance on the cross-validation folds. APR: area under the precision-recall curve; MEWS: Modified Early Warning Score; qSOFA: quick SOFA; SIRS: systemic inflammatory response syndrome.

The comparison of *InSight* results with each of qSOFA, MEWS, and SIRS, as well as the SOFA and SAPS II scores computed at ICU admission, for sepsis onset and preceding times are presented graphically in Figures 3, 4, and 5. Additional performance measures appear in Table 4. At the time of onset, the *InSight* AUROC (0.8799 [SD 0.0056]) and APR are superior to all of the other methods tested (*P*<.001 in all cases, assuming normality). This advantage persists at longer preonset prediction times (*P*<.001 for all AUROC cases and precision-recall for methods other than SOFA; *P*<.001 and *P*=.37 for APR against SOFA at 1 and 2 hours before onset, inferior to admission SOFA in APR with *P*=.001 and *P*=.009 for 3 and 4 hours before onset).

The ROC curves of *InSight* and the competing scores are shown in Figure 3. As *InSight* is trained to value sensitivity and specificity equally, the ROC curves tend to show a balance between these two constraints. The AUROC advantage held by *InSight* is demonstrated by the form of the ROC curve compared with the other methods (ie, the *InSight* ROC curve generally shows higher sensitivity or specificity, or both, compared with points on the other curves).

Figure 5 shows the area under the precision-recall curves for all scores. precision-recall and ROC curves have a one-to-one correspondence, but emphasize different aspects of the data. While ROC curves are not sensitive to the prevalence of the Class 1 condition (ie, sepsis), the precision value (also known as positive predictive value or PPV) is directly influenced by the prevalence of the Class 1 condition. Further performance measures are presented in Table 4. *InSight* simultaneously achieves moderate sensitivity and specificity, while also attaining good diagnostic odds ratio (DOR) values.

We performed an auxiliary analysis where we eliminated patients who received antibiotics prior to the start of their ICU stay, and the resulting AUROC and model performance metrics were not found to be significantly different from those reported in Figure 3 and Table 4.

**Figure 6:**
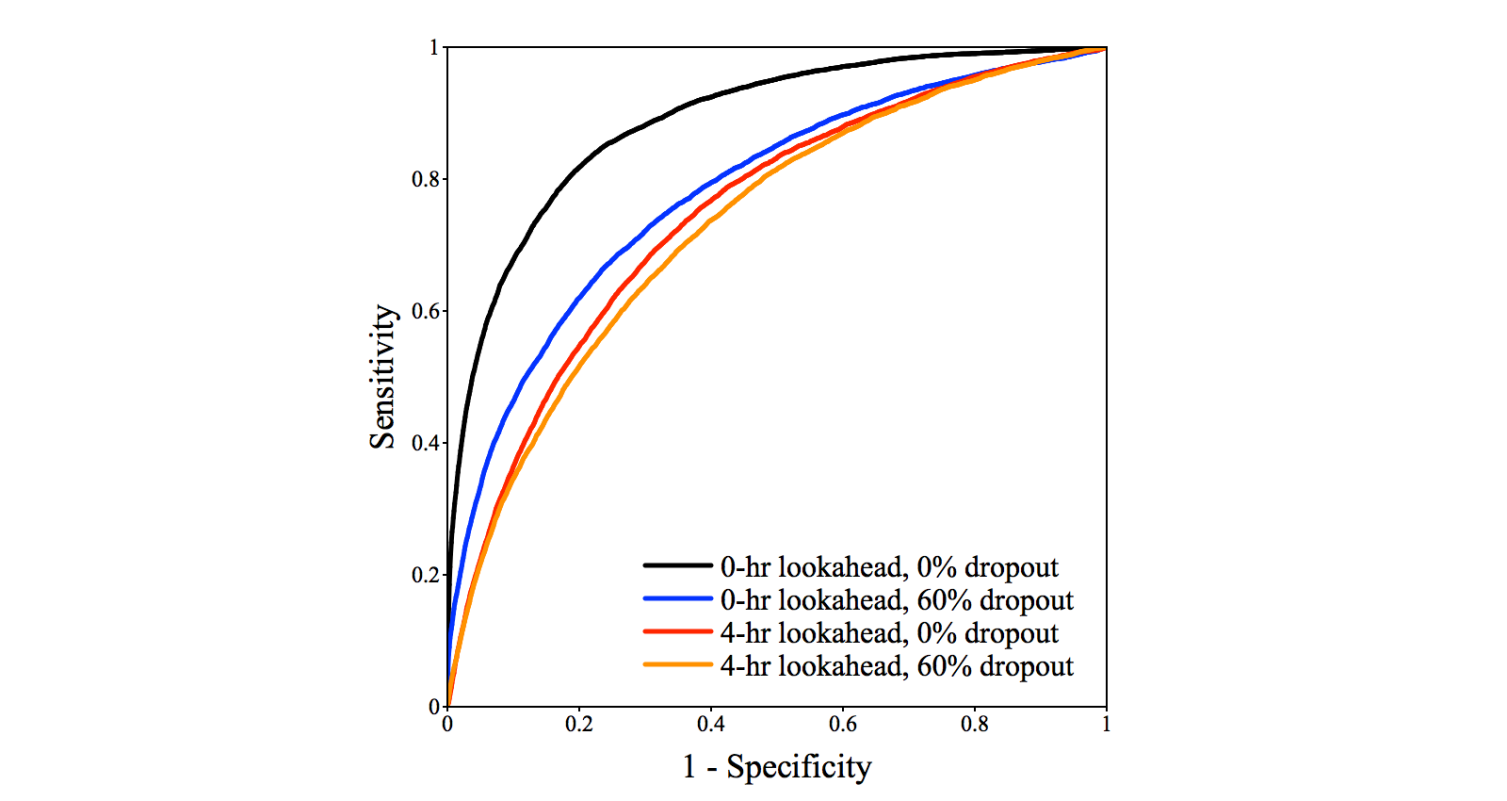
Receiver operating characteristic curves for *InSight* at selected preonset prediction times and random dropout frequencies.

**Figure 7:**
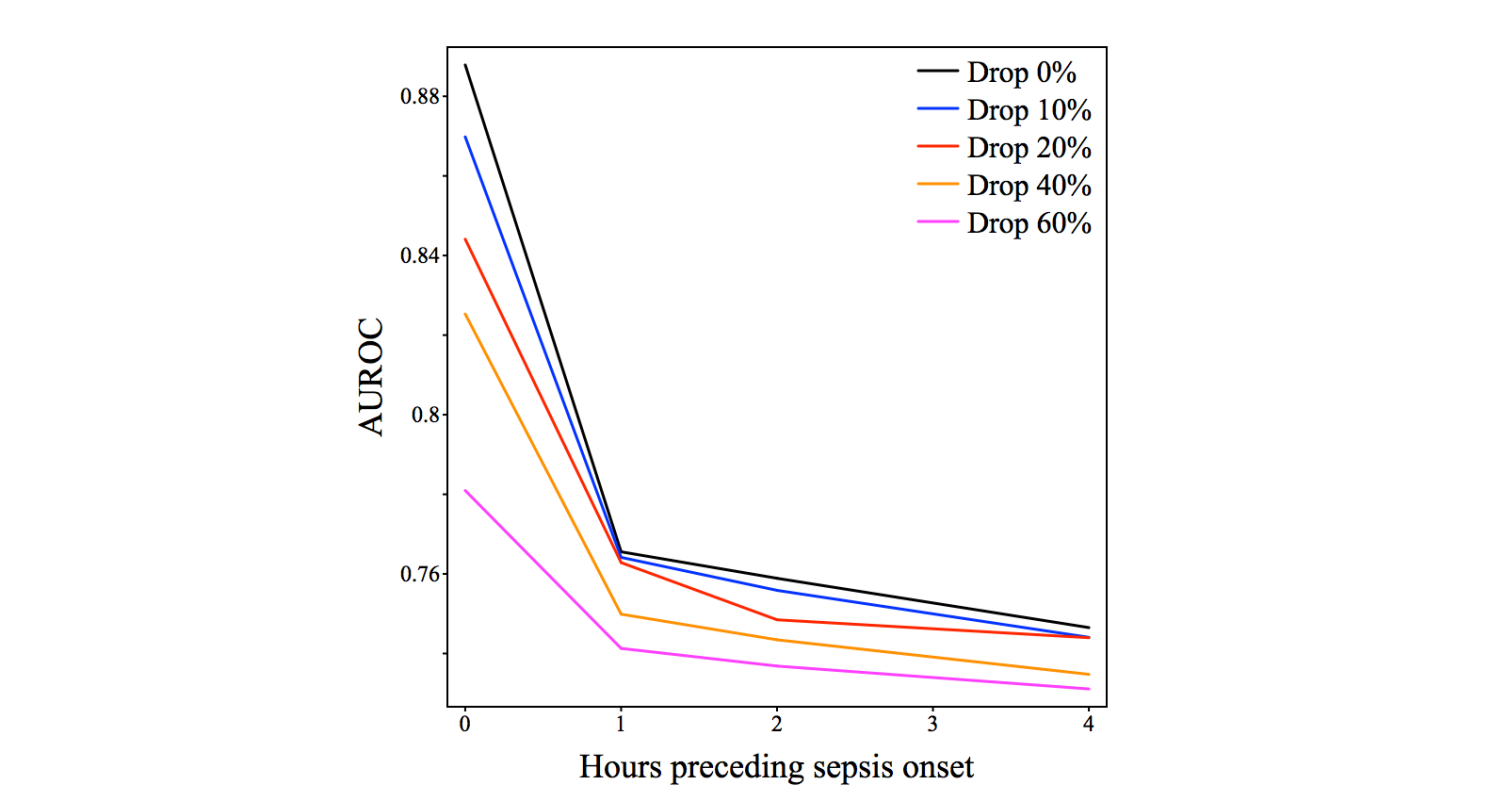
Area under the receiver operating characteristic curve (AUROC) for *InSight* versus preonset prediction time. Each line corresponds to the indicated measurement dropout frequency. All experiments are run with 4-fold cross-validation, with the data repartitioned 4 times.

**Figure 8:**
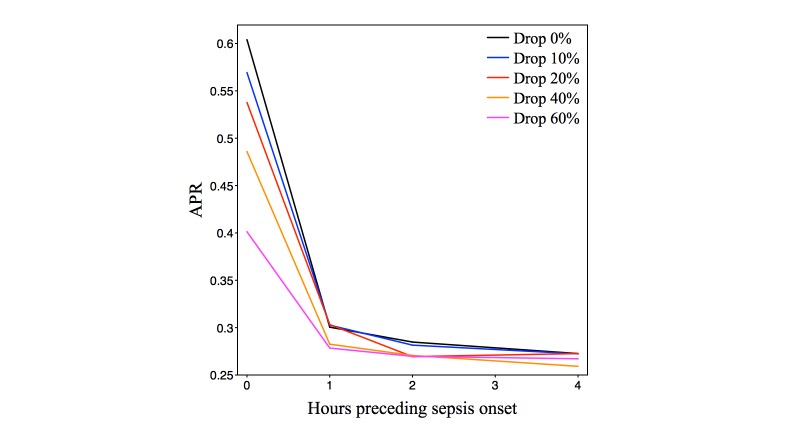
Area under the precision-recall curve (APR) for *InSight* versus preonset prediction time. Each line corresponds to the indicated measurement dropout frequency. All experiments are run with 4-fold cross-validation, with the data repartitioned 4 times.

We computed the performance of the *InSight* system for random observation deletions, where these occurred with probability *P* = {0, .1, .2, .4, and .6}, with preonset prediction times of 0, 1, 2, and 4 hours. The results of these experiments appear as Figures 6,7, and 8 and Table 5. The typical frequencies of raw data in our patient population (Table 3) are approximately one per hour. Since we discretize time in one-hour intervals, the random data deletions studied here are in a critical regime around the discretization rate and should be expected to affect *InSight’s* performance.

Figure 6 shows the ROC curves at selected preonset prediction times and random dropout frequencies. The ROC curves largely maintain performance, even with more than half of all measurements removed. In fact, for predictions 4-hours ahead, and with 60% of measurements missing, *InSight* achieves performance similar to qSOFA detection with no dropout. Full area under ROC and precision-recall curves as a function of time preceding onset are illustrated in Figures 7 and 8, and are further detailed in Table 5.

**Table 4:** Detailed performance measures for *InSight* and competing scores on the complete Multiparameter Intelligent Monitoring in Intensive Care version III (MIMIC-III) data set, with operating points chosen to make sensitivities close to 0.80. Note that all of quick SOFA’s operating points produced sensitivities far from 0.80.

	*InSight*: 0 hours	*InSight*: 4 hours	SIRS^a^	quick SOFA	MEWS^b^	SAPS II^c^	SOFA^d^
AUROC^e^	0.88 (SD 0.006)	0.74 (SD 0.010)	0.61	0.77	0.80	0.70	0.73
APR^f^	0.60 (SD 0.016)	0.28 (SD 0.013)	0.16	0.28	0.33	0.23	0.28
Sensitivity	0.80	0.80	0.72	0.56	0.70	0.75	0.80
Specificity	0.80	0.54	0.44	0.84	0.77	0.52	0.48
F1^g^	0.47	0.30	0.24	0.39	0.40	0.27	0.27
DOR^h^	15.51	4.75	2.06	6.33	7.85	3.26	3.71
LR+^i^	3.90	1.75	1.30	3.37	3.05	1.57	1.55
LR-^j^	0.25	0.37	0.63	0.53	0.39	0.48	0.42
Accuracy	0.80	0.57	0.47	0.80	0.76	0.55	0.52

^a^SIRS: systemic inflammatory response syndrome

^b^MEWS: Modified Early Warning Score.

^c^SAPS II: Simplified Acute Physiology Score II.

^d^SOFA: Sequential (Sepsis-Related) Organ Failure Assessment.

^e^AURUC: area under the receiver operating characteristic curve.

^f^APR: area under the precision-recall curve.

^g^F1: harmonic mean of precision and recall.

^h^DOR: diagnostic odds ratio.

^i^LR+: positive likelihood ratio.

^j^LR-: negative likelihood ratio.

**Table 5:** Detailed performance measures of *InSight* when tested and trained with raw data dropouts. Operating points were chosen according to the same procedure as in Table 4.

	*InSight*, 0 hour, 0% dropout	*InSight*, 0 hour, 10% dropout	*InSight*, 0 hour, 20% dropout	*InSight*, 0 hour, 40% dropout	*InSight*, 0 hour, 60% dropout	*InSight*, 4 hour, 0% dropout	*InSight*, 4 hour, 60% dropout
AUROC^a^	0.89 (SD 0.010)	0.87 (SD 0.006)	0.84 (SD 0.011)	0.83 (SD 0.012)	0.78 (SD 0.013)	0.75 (SD 0.008)	0.73 (SD 0.010)
APR^b^	0.60 (SD 0.022)	0.57 (SD 0.015)	0.54 (SD 0.022)	0.49 (SD 0.021)	0.40 (SD 0.015)	0.27 (SD 0.012)	0.27 (SD 0.009)
Sensitivity	0.80	0.80	0.80	0.80	0.80	0.80	0.80
Specificity	0.82	0.78	0.72	0.68	0.59	0.55	0.52
F1^c^	0.49	0.45	0.40	0.37	0.32	0.30	0.29
DOR^d^	17.90	14.14	10.23	8.31	5.76	4.95	4.38
LR+^e^	4.37	3.62	2.85	2.46	1.95	1.79	1.67
LR-^f^	0.24	0.26	0.28	0.30	0.34	0.36	0.38
Accuracy	0.82	0.78	0.73	0.69	0.61	0.58	0.55

^a^AUROC: area under the receiver operating characteristic curve.

^b^APR: area under the precision-recall curve.

^c^F1: harmonic mean of precision and recall.

^d^DOR: diagnostic odds ratio.

^e^LR+: positive likelihood ratio.

^f^LR-: negative likelihood ratio.

## Discussion

### Principal Findings

We tested and validated *InSight,* a machine learning-based system for predicting the onset of sepsis from flexible and minimal data. Using the retrospective MIMIC-III dataset and the new Sepsis-3 definition of sepsis, we trained this system to predict sepsis onset and tested its performance. *InSight* classifies patients (septic vs nonseptic) with a performance that is superior to the corresponding qSOFA, SIRS, and MEWS scores, and it is also superior to the SOFA and SAPS II scores generated at time of admission based on AUROC analysis. It is important to note that MEWS and SAPS II were not explicitly designed for the purpose of sepsis-related severity measurement or prediction. However, these canonical scores represent an important and well-known benchmark for comparison since they are commonly used for sepsis management in clinical settings. *InSight’s* superior performance is achieved despite using only age and extended vital sign measurements. All of the extended vital sign measurements (systolic blood pressure, pulse pressure, respiration rate, heart rate, SpO_2_, body temperature, and GCS) are commonly available and are easily assessed at the bedside. While the *InSight* system does not offer a manually computable score, it does provide a compelling alternative to the qSOFA and SIRS scores in an increasingly EHR-integrated hospital environment.

Figures 3 and 4 compare the ROC curves of *InSight* with alternative scoring systems. *InSight* generally attains significantly better performance. This result means that, for nearly any specified sensitivity, *InSight* offers superior specificity, and vice versa. Under the gold standard defined above, sepsis has a prevalence of 11.3% (2577/22,853). Furthermore, removing patients who received pre-ICU antibiotics from the analysis did not significantly affect the results. As seen in the precision-recall curve of Figure 5, *InSight’s* PPV can easily be operated over 0.5 for 0-hour detection. For prediction one or more hours ahead, a PPV of approximately 0.4 can be obtained if a relatively low sensitivity is acceptable. This would potentially allow narrowly targeted interventions to be applied to a subset of patients whose sepsis diagnosis is nearly certain, while identifying the remaining cases in a more timely manner when their impending sepsis onset becomes more evident.

The detailed numerical results in Table 4 show that *InSight* provides a superior sepsis predictor compared with the alternatives, which tend to have average performance across all measures (SAPS II, MEWS, SOFA) or a large imbalance between sensitivity and specificity (qSOFA, SIRS). While we could choose a different alarm threshold to match or exceed the sensitivity of qSOFA, we would do so at the cost of the other metrics. With respect to the competing scores, the performance of *InSight* stands out, both because it has a high DOR and because it strikes a balance between the other performance metrics without degrading another area. Unlike accuracy, DOR is independent of the prevalence of the positive class. Notably, *InSight* performance 4 hours prior to the onset of sepsis is at least as strong, if not stronger, than the comparison methods.

To improve performance over current scoring systems, *InSight* learns patterns in the trends and correlations among extended vitals through a machine learning process. Several of these extended vitals are also used by SIRS and qSOFA, in conjunction with a suspicion of infection, to diagnose for sepsis, especially outside the ICU setting. The use of correlations in *InSight* is an extension of the approach used by the MEWS scoring system that normalizes patient vitals and sums the results, thereby incorporating some interrelations among different clinical variables. APACHE III also incorporates interrelations among certain variables (eg, pH and pCO_2_) via lookup tables. Similarly, the use of trend information in *InSight* builds on the strategy used by SOFA and APACHE III, where the highest daily value of several patient measurements may be used for score calculations, which implies incorporation of some temporal information.

*InSight* is also shown by these experiments to be relatively resistant to performance loss from reduced measurement availability. Table 5 presents a variety of performance data for predictors throughout a range of preonset prediction time and random dropout frequency. *InSight* at 40% dropout frequency and at the time of sepsis onset (Table 5) attains performance superior to MEWS at the time of sepsis onset (Table 4). Even with a 60% dropout frequency, *InSight* attains performance that is slightly better than at a prediction time 4 hours before sepsis onset. This result indicates that even if measurement frequency is reduced to well below the prevailing temporal discretization frequency, prognostication is a more difficult task than dealing with measurement dropout. Figure 6, which shows individual ROC curves, and Figures 7 and 8, which show trends across the regime and inter-fold variability, also support this conclusion.

These experiments show *InSight* to be an effective, high performance predictor that uses readily available bedside data for its calculation. This performance is achieved by applying machine learning methods to the relatively simple vital signs data. As noted in the methods section, *InSight* only uses data that would be readily available via ubiquitous monitoring devices (pulse oximeter, blood pressure monitor, etc) and a simple exam. This is a significant difference when compared with the MEWS, SOFA, and SAPS II scoring systems. Additionally, because *InSight* is a machine learning algorithm, it is not restrained to these particular input measurements. In implementation, *InSight* can be trained on the data available in any given setting and will utilize the available measurements that are most relevant to the desired prediction outcome. Of course, performance metrics would be expected to vary with the type and amount of input data available, and training and validation would be required on any novel dataset.

While this is a retrospective study, we are planning future prospective studies through EHR integration of the *InSight* algorithm in an ICU setting. Within that setting, *InSight* has the potential to identify patients at risk of developing sepsis prior to serious patient deterioration or multiple organ failure. *InSight*'s predictive discrimination at 4 hours preceding sepsis onset, as demonstrated in this work, may afford a valuable time window for course-altering clinical intervention. Furthermore, the improvement of sensitivity and specificity over existing sepsis detection methods increases confidence in the accuracy of the *InSight* sepsis alert and therefore may reduce the “alarm fatigue” associated with inaccurate warning systems [17]. Alarm fatigue is defined as the scenario in which too many alarms lead to a decrease in clinician response speed or rate. With increased accuracy and advance warning of impending sepsis, *InSight* has the potential to improve monitoring and treatment for patients who are at risk of sepsis development and to reduce the associated high rates of morbidity and mortality.

Many scoring systems are used for predicting patient outcomes or treatment guidance, despite not being developed for these purposes (eg, SOFA). We present a purpose-built alternative to these systems, based on ubiquitously available vital sign data, for predicting sepsis onset in ICU patients. In this study, *InSight* outperforms all of the other sepsis scoring systems during testing in a variety of realistic conditions. Compared with previous machine learning systems, *InSight* attains similar [18] or better [19,20] AUROC performance at sepsis detection (0.8799 [SD 0.0056], at 0-hours preonset) and offers some prognostic ability while using a significantly more limited collection of patient data [21].

### Limitations

There are several practical limitations in this study. First, it is not designed to “discover” a set of rules that could create a manual scoring system. *InSight* is designed as an automatic, EHR-integrated system. Due to its several sequential calculations, including mapping of the input data to a higher-dimensional feature space, *InSight* scores are infeasible to calculate by hand. These calculations are trivial for a computer, however, and can be executed in fractions of a second. Future work may investigate how the *InSight* system can provide clear explanations of its predictions to clinicians including formulae for approximate manual calculations. The gold standard that is based on the Sepsis-3 definitions [3] also presents several difficulties. Sepsis onset is a poorly defined event and identification of an onset time was not the intention of Singer et al; therefore, using their definition for this purpose may be problematic.

We have also chosen to use only a subset of patients in the MIMIC-III (v1.3) database. Because the currently available version of MIMIC-III under-reports cultures, particularly for patients recorded using the CareVue system, we have chosen to work only with patients recorded using the alternative Metavision system to get a more complete picture of suspected infection at various sites. Future work will address these limitations.

An additional limitation is that this study was performed exclusively on ICU data and at a single center, which may limit generalization of our results to other hospitals and hospital systems. While *InSight* operates using only data that are commonly available in nonICU wards, the outcomes reported in this particular study on ICU data do not provide a guarantee of equivalent performance in other settings.

### Conclusion

Sepsis prediction is a challenging problem and remains so despite many years of research and development efforts because its manifestation is often unclear until later stages. *InSight* is a machine learning approach specifically designed for this challenge. In this study, *InSight* is shown to be an effective predictor that uses simple and readily available patient data for its calculation. However, in our experiments, the performance of *InSight* is better than the complex, laboratory-value-dependent SAPS II and SOFA scores when computed at ICU admission, and it performs comparably with other machine learning methods in the literature without requiring the laboratory tests that they incorporate. These experiments also show that *InSight* is resistant to performance degradation from significant random data deletion used to simulate real-world data unavailability. *InSight* is also superior in performance to the qSOFA and SIRS scoring systems that use similar data for calculation. While these two scores have the advantage of being easily computable without computer assistance, *InSight* is readily applicable autonomously in an EHR-integrated environment and offers a high-performance alternative without requiring the collection of any additional data.

## Abbreviations

APR: area under the precision-recall curve
AUROC: area under receiver operating characteristic
BIDMC: Beth Israel Deaconess Medical Center
CCU: coronary care unit
CSRU: cardiac surgery recovery unit
DiasABP: invasive diastolic arterial blood pressure
DOR: diagnostic odds ratio
EHR: electronic health records
F1: harmonic mean of precision and recall
GCS: Glasgow Coma Score
HIPAA: Health Insurance Portability and Accountability Act
ICU: intensive care unit
IQR: interquartile range
LR+: positive likelihood ratio
LR-: negative likelihood ratio
MEWS: Modified Early Warning Score
MICU: medical intensive care unit
MIMIC III: Multiparameter Intelligent Monitoring in Intensive Care version III
NIDiasABP: noninvasive diastolic arterial blood pressure
NISysABP: noninvasive systolic arterial blood pressure
NPV: negative predictive value
PPV: positive predictive value
qSOFA: quickSOFA
ROC: receiver operating characteristic
SAPS II: simplified acute physiology score II
SICU: surgical intensive care Unit
SIRS: systemic inflammatory response syndrome
SOFA: Sequential (Sepsis-Related) Organ Failure Assessment
SpO2: peripheral capillary oxygen saturation
SysABP: invasive systolic arterial blood pressure
TSICU: trauma-surgical intensive care unit
